# Design, analysis, and demonstration of the COAST guidewire robot with middle tube rotation for endovascular interventions

**DOI:** 10.1038/s41598-024-75871-7

**Published:** 2024-11-12

**Authors:** Sharan R. Ravigopal, Revanth Konda, Nidhi Malhotra, Jaydev P. Desai

**Affiliations:** Medical Robotics and Automation (RoboMed) Laboratory, Wallace H. Coulter Department of Biomedical Engineering, Georgia Institute of Technology, Atlanta, GA 30332 USA

**Keywords:** Biomedical engineering, Mechanical engineering

## Abstract

Minimally invasive procedures for endovascular interventions involve manual navigation of a guidewire. Endovascular interventions encompassing highly tortuous vessels would benefit from guidewires which exhibit higher dexterity. This paper introduces a version of the COAST (COaxially Aligned STeerable) guidewire system capable of exhibiting higher dexterity. The system presented in this paper consists of three coaxially aligned tubes with a tendon to actuate the middle tube. Furthermore, it is possible to independently rotate the middle tube with respect to the outer tube. This variation enables the guidewire to achieve curvature in different planes while avoiding rotation of the entire structure. We also present the simulated stability of the guidewire with different outer tube geometries and experimentally validate the model. Experimental analysis and modeling of the kinematic behavior of the system is presented. A model to calculate the curvature vs. tendon stroke relationship for the optimal notch geometry is presented with an average RMSE of 0.16 mm. A control strategy addressing the snapping instabilities to ensure reliable operation is discussed. A custom phantom vessel and an aortic arch phantom model were used to demonstrate the ability of the system to safely navigate through tortuous pathways without exhibiting these elastic instabilities.

## Introduction

The navigation of intricate and narrow blood vessels using traditional guidewires is heavily dependent on the expertise of interventional cardiologists. The difficulty in manipulating the distal end of the guidewire poses a risk of blood vessel perforation^1^. In contrast, robotically steerable guidewires offer the advantage of remote control with superior precision, granting operators enhanced dexterity and control over their movements^2^. This heightened level of accuracy proves particularly crucial in scenarios involving constrained vasculature and multiple bifurcations, where the susceptibility to accidental vessel damage and incorrect guidewire placement is elevated^3^. Robotically steerable guidewires and catheters can utilize magnetic actuation^4^, thermal actuation^5^, hydraulic-chamber based actuation^6^, tendon-driven mechanisms (TDMs)^7–10^ and concentric tube mechanisms^11^.

The COaxially Aligned STeerable (COAST) guidewire robot^12–15^ that was previously developed in our laboratory is an example of tendon-driven multi-tube structure capable of varying the bending length. It consists of three tubes: an outer, middle, and inner tube. Previously^16^, the outer and middle tubes were machined with unidirectional asymmetric notch patterns to enable directional bending. The notches of the middle tube and outer tube were phased exactly $180^\circ$ apart so that the structure would bend in the direction opposite to the notches of the outer tube. This guidewire system has been fabricated with an outer diameter as small as 400 $\upmu$m. There has been work in intrinsic shape sensing^7^, kinematic analysis^17^, and closed-loop control^12^ for the COAST guidewire. Although robotic guidewire systems like the COAST guidewire can efficiently navigate through relatively tortuous vasculatures^15^, navigation in branches which are outside of the plane of bending of the guidewire requires the entire structure to be rotated such that the guidewire is suitably aligned with the vessel under consideration^18^. This mechanism may not be ideal, particularly in scenarios where the vessels are confined. Furthermore, when navigating guidewires through tortuous vasculature, rotational motion with respect to the vessel may cause vessel damage and perforation^19,20^.

A popular actuation method for surgical robots is the concentric tube mechanism (CTM), which consists of multiple concentric tubes^21^ with generally fixed pre-curvatures. Each tube within the robotic structure can be actuated relative to one another through linear and rotational motions^22^, which results in a change in the overall curvature of the combined tube structure^23^. More specifically, CTMs utilize rotational motion of tubes with respect to each other to vary the bending plane of the combined robot structure. This type of actuation system can potentially be leveraged to control the angle of the plane of bending of the COAST guidewire. More specifically, a system that can only rotate the middle tube of the guidewire can be useful to increase the overall range of rotation of the guidewire to navigate tighter vasculatures. However, a method to control the angle of the plane of bending of the middle tube of the robot separate from the remainder of the robot is an open problem.

In this work, the COAST guidewire equipped with independent middle tube rotational motion is presented. The middle tube is tendon-actuated and has the ability to rotate independently with respect to the outer tube, and hence the rest of the robot body. Unlike other concentric-tube robots, this design can actively vary the curvature of the inner tube like in TDMs, and unlike conventional TDMs, the middle tube of the robot can be rotated. Independent middle tube rotation enables omnidirectional guidewire bending without the need to rotate the entire three-tube structure. This mechanism can thus potentially eliminate the risk of perforation or rupture in tortuous vasculature^3^. This design innovation can potentially facilitate traversal through more distally curved segments, particularly in tighter bifurcations. The primary contributions of this work are:

1. Integrating middle tube rotation capabilities to the COAST guidewire robot system.
2. Analysis of the stability and kinematic behavior of the robot with different outer tube designs at different middle tube rotation angles, bending lengths, and different curvatures of the robot.
3. Development of a kinematic model for the curvature–tendon stroke relationship for a guidewire at different middle tube rotation angles.
4. Navigation through a custom phantom model and an aortic arch phantom model exhibiting varying curvatures and a bifurcation.

Previously, we devised a preliminary setup that can rotate the middle tube of the COAST guidewire system^24^. The enhancements of this paper over our previous work^24^ include:

1. Stability analysis of the guidewire with several notch designs for the outer tube through simulation and experiments.
2. Experimental analysis of the kinematic behavior of the guidewire with different notch designs for the outer tube.
3. Modeling and experimental verification of the kinematic behavior of the guidewire with middle tube rotation.
4. Demonstration of the middle tube rotation in a different custom vascular phantom and an aortic arch phantom model using a novel control strategy developed from experimental analysis of the kinematic behavior.

## Results

### COAST guidewire with middle tube rotation

**Fig. 1:**
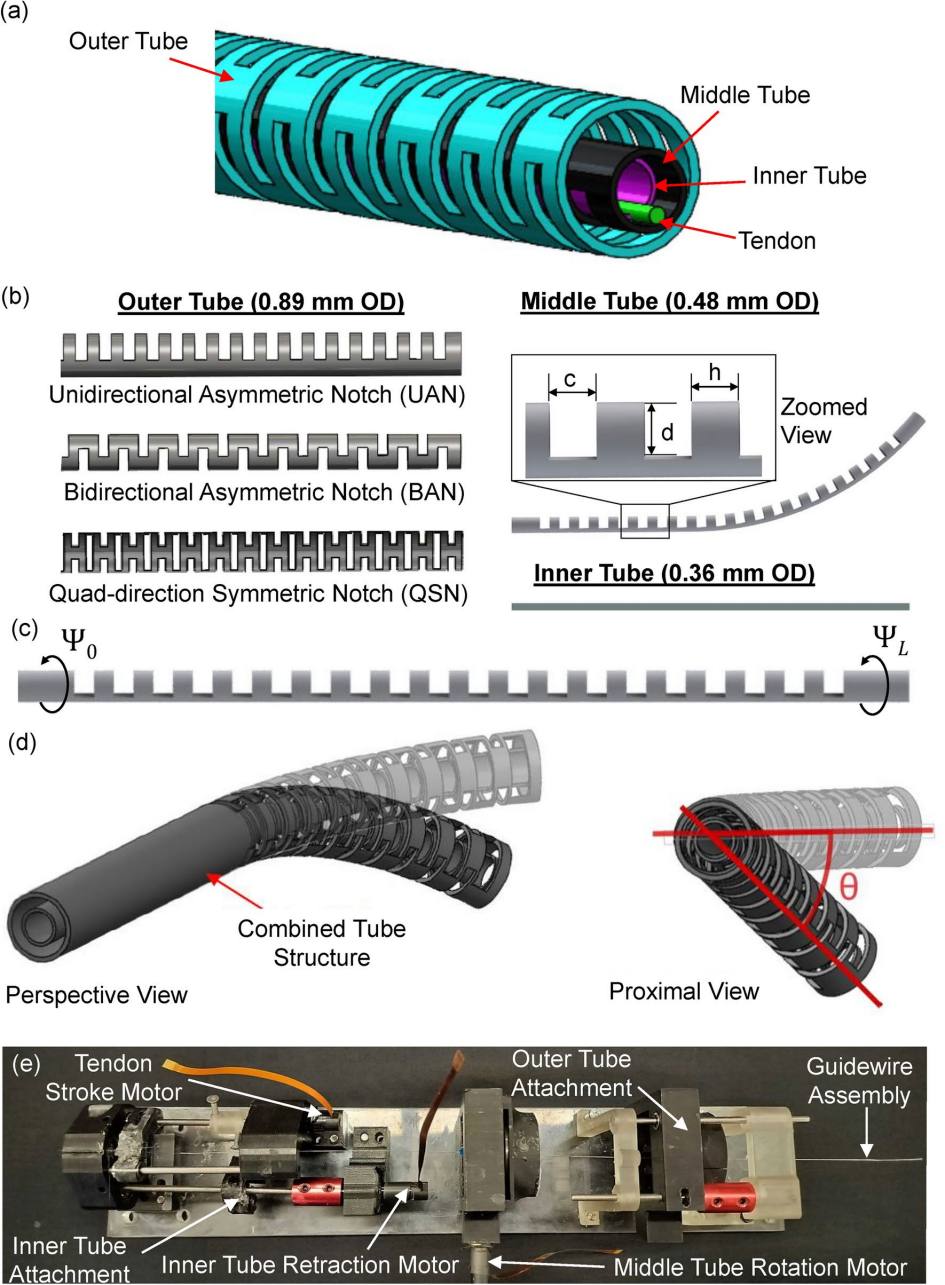
(**a**) Rendering of the COAST guidewire with the inner, middle, and outer tubes, and the tendon. (**b**) Illustration of the individual tubes with their respective notch patterns and outer diameters. (**c**) Illustration of the middle tube rotation parameters, $\psi _0$, the proximal rotation angle, and $\psi _L$, the distal rotation angle. (**d**) Illustration of the combined tube structure and the angle of the plane of bending, $\theta$, with perspective and proximal views. (**e**) Image of the actuation system and its motors used to rotate the middle tube, insert/retract the inner tube, and pull the tendon of the COAST guidewire system.

The COAST guidewire robot used in this work consists of three super-elastic nitinol tubes (Edgetech®, Miramar, FL, USA): an inner tube, middle tube, and outer tube with outer diameters (OD) of 0.36 mm, 0.48 mm, and 0.89 mm, respectively, as shown in Fig. 1(a). The middle tube of the guidewire robot was machined with a unidirectional asymmetric notch (UAN) pattern using a femtosecond laser (WS-Flex Ultra-Short Pulse Laser Workstation, Optec®, Frameries, Belgium). Due to the UAN pattern, the machined middle tube develops a pre-curvature due to asymmetric heating during the laser micromachining process. The UAN pattern machined into the middle tube is characterized by the notch depth, *d*, the notch spacing, *c*, and the notch width, *h* as shown in Fig. 1(b). These design parameters are used to increase the compliance of the joints in a single bending plane. Multiple outer tubes with varying notch patterns were fabricated to find an optimal design for realizing the COAST guidewire robot with middle tube rotation capabilities. The notch patterns, shown in Fig. 1(b), are a UAN pattern, bidirectional asymmetric notch (BAN) pattern, and a quad-directional symmetric notch (QSN) pattern. The BAN pattern comprises two UAN patterns aligned $180^\circ$ apart so that the preferential bending is in both the $0^\circ$ and $180^\circ$ directions. Due to the symmetry of the BAN and QSN patterns, the outer tubes with those notch patterns had a negligible pre-curvature which can be assumed to be zero. Meanwhile, the UAN joint had a designated pre-curvature that was formed from machining the tubes, resulting from asymmetric heating. The guidewire parameters constitute among other things, the notch parameters, the number of notches, the inner and outer diameter of each tube, the length of each tube, the length and diameter of the tendon, and the material of the tubes and tendon.

The middle tube was attached with a nitinol tendon of OD 0.1 mm such that pulling on the nitinol tendon at the proximal end will bend the guidewire. Additionally, an unmachined inner tube was inserted into the middle tube. The retraction and insertion of the inner tube would increase or decrease the bending length of the middle tube, respectively. The Young’s Modulus, *E*, of the nitinol tubes, is given by the manufacturer as $\approx$ 40–45 *GPa*. The parameters for each component of this COAST guidewire robot are summarized in Table 1 and Table 2.

**Table 1:** Specifications of the COAST guidewire prototype tubes.

Items	Outer tube	Middle tube	Inner tube	Tendon
Length of the notched section (mm)	50	50	–	–
Outer diameter, 2$r_o$, (mm)	0.89	0.48	0.36	0.10
Inner diameter, 2$r_i$, (mm)	0.77	0.40	0.27	–
Young’s modulus, *E*, (GPa)	40–45	40–45	40–45	40–45

The introduction of the rotational motion of the middle tube required an outer tube with a larger outer diameter of 0.89 mm as compared to the 0.4 mm tube used in the previous work^16^. This was done to provide sufficient clearances (0.14 mm) between the outer tube and the middle tube to enable smooth rotational motion of the middle tube when no tendon stroke was applied. A relatively smaller outer tube which provided a clearance of 0.04 mm, resulted in the non-smooth rotation of the middle tube due to insufficient clearances and high friction.

The middle tube rotation allowed the system to achieve higher dexterity as compared to the traditional COAST guidewire system: The traditional guidewire system had UAN notches aligned at a fixed $180^\circ$ phase difference between the outer and the middle tubes. In contrast, for the system presented in this work, the alignment of the notches of the middle tube can be actively controlled. This ensured that the plane of bending could be varied while not exerting excessive work on the vessel in which the guidewire is placed. The control variables of the presented system are:

$$\begin{aligned} \begin{bmatrix} \psi \\ X_1 \\ X_2\\ X_3\\ X_4\\ \end{bmatrix} =\begin{bmatrix} \text {Middle Tube Rotation Angle (}^{\circ }\text {)}\\ \text {Stroke of Tendon (}\text{mm}\text {)}\\ \text {Stage Displacement (}\text{mm}\text {)}\\ \text {Inner Tube Retraction (}\text{mm}\text {)}\\ \text {Outer Tube Extension (}\text{mm}\text {)} \end{bmatrix} \end{aligned}$$

### Guidewire actuation system

A motorized actuation system (Fig. 1(e)) was used to steer the robotic guidewire. The proximal end of the outer tube was held in a module located in the front of the actuation system. The middle tube was fixed to the middle tube rotation system, which consisted of a brushed DC motor to rotate the middle tube with respect to the outer tube. The tendon stroke was controlled by a module in the rear end of the actuation system. The inner tube displacement was also controlled by a module in the rear end of the actuation system. The insertion and retraction of the inner tube could be controlled to modify the bending length of the guidewire system. The actuation mechanisms described above were achieved by precise brushed DC motors (Maxon Group, Sachseln, Switzerland). The tendon stroke was achieved through a motor attached to a lead screw. The stage displacement was actuated by a brushed DC motor (Pololu, NV, USA) attached to a lead screw that allows for the insertion and retraction of the actuation stage.

For the rotational assembly of the middle tube, a brushed DC motor connected to a bevel gear train was utilized. At the center of the bevel gear and in the outer tube module, openings were designed to snap-fit 3D-printed collets. The middle tube was secured to a 3D-printed collet positioned at the center of the corresponding bevel gear, allowing for the rotation of the middle tube. The outer tube was secured to a collet positioned in its module at the front of the actuation system. Reference commands for each motor were sent over bluetooth to a Simulink® Desktop Real-Time™ application (Simulink® 2023a, MathWorks Inc., Natick, MA, USA) using a remote controller at a frequency of 500 Hz. Each motor was controlled using a proportional-integral-derivative (PID) controller with control signals transmitted to a Teensy microcontroller (PJRC, OR, USA) using user datagram protocol (UDP).

### Stability analysis

#### Simulation results

**Fig. 2:**
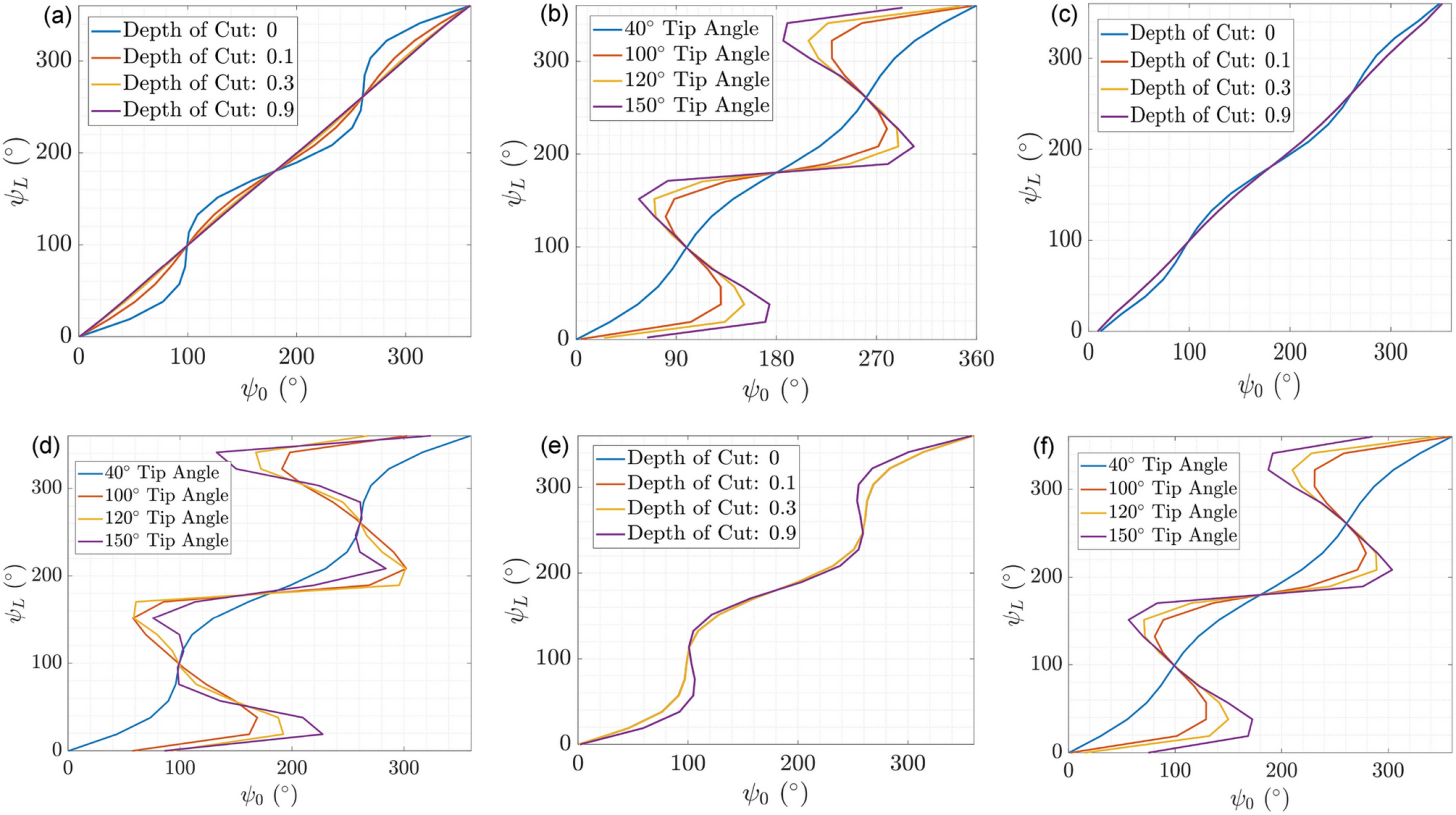
(**a**) Proximal angle–distal angle correlation using outer tube with UAN pattern for differing depths of cut for the outer tube. The tip angle of middle and outer tubes was $60^\circ$. (**b**) Proximal angle–distal angle correlation using outer tube with UAN pattern with differing curvatures. The tip angle varies from $40^\circ$ to $150^\circ$. (**c**) Proximal angle–distal angle correlation using outer tube with BAN pattern for differing depths of cut for the outer tube. The tip angle of middle and outer tubes was $60^\circ$. (**d**) Proximal angle–distal angle correlation using outer tube with BAN pattern with differing curvatures. The tip angle varies from $40^\circ$ to $150^\circ$. (**e**) Proximal angle–distal angle correlation using outer tube with QSN pattern for differing depths of cut for the outer tube. The tip angle of middle and outer tubes was $60^\circ$. (**f**) Proximal angle–distal angle correlation using outer tube with QSN pattern with differing curvatures. The tip angle varies from $40^\circ$ to $150^\circ$.

We simulated the proximal angle ($\psi _0$) to distal angle ($\psi _L$) mapping by varying the tube curvatures, the depths of cut for the notches, and the notch geometry for the outer tube (see Fig. 1(b),(c)). We would like to draw a distinction between the elastic instabilities caused by friction and the elastic instabilities that are caused by the interaction of tubes with high pre-curvatures. The elastic instabilities that are caused by the design of the tubes themselves are investigated and simulated in this study. The design parameters of the middle tube were constant and are detailed in Table 2. The $\psi _0$–$\psi _L$ correlations for the robot with an outer tube with UAN pattern and varying depths of cut are shown in Fig. 2(a). The regions where a value of $\psi _0$ generates more than one value of $\psi _L$ indicate unstable regions of the system. The results demonstrate that increasing the depth of cut for the outer tube stabilizes the system. This is because increasing the depth of cut results in a decrease in the stiffness of the outer tube which contributes to stability^25^.

**Table 2:** Notch specifications of the middle and outer tubes.

Items	Notch depth *d* (mm)	Notch width *h* (mm)	Notch spacing *c* (mm)
Middle tube UAN pattern	0.27	0.30	0.30
Outer tube UAN pattern	0.54	0.30	0.30
Outer tube BAN pattern	0.54	0.30	0.30
Outer tube QSN pattern	0.52	0.20	0.20

The $\psi _0$–$\psi _L$ correlations with an increase in the curvature of both the tubes with a constant bending length, from $40^\circ$ tip angle to $150^\circ$ tip angle is shown in Fig. 2(b). The results demonstrate that increasing the curvature of both tubes decreases the stability of the system^25^. A similar analysis is conducted for the outer tube with BAN and QSN patterns, as shown in Fig. 2(c)–(f). Both cases show a similar trend of increasing the depth of cut increases stability for these notch geometries and that increasing the curvatures would decrease the stability of the system.

These simulations illustrate the conventional input-to-output mapping of the middle tube rotation angle for several notch parameter designs, which have tubes with different curvatures and rigidities. These graphs can be shown to find the regions of instabilities in the design space, which occur where the regions of this plot have a negative slope. The graphs that have only a positive slope have stable configurations throughout the range of $\psi _0$, which shows that it is difficult to achieve sufficient stability around the region where $\psi _0 = 90^\circ$ and $\psi _0 =270^\circ$ for all three notch patterns. As shown in^25^, decreasing the $\zeta _{j}$ value (Eq. (12)) for the tubes makes the stability greater, which is what we achieve while machining the nitinol tubes to fabricate these COAST guidewires.

#### Experimental results

**Fig. 3:**
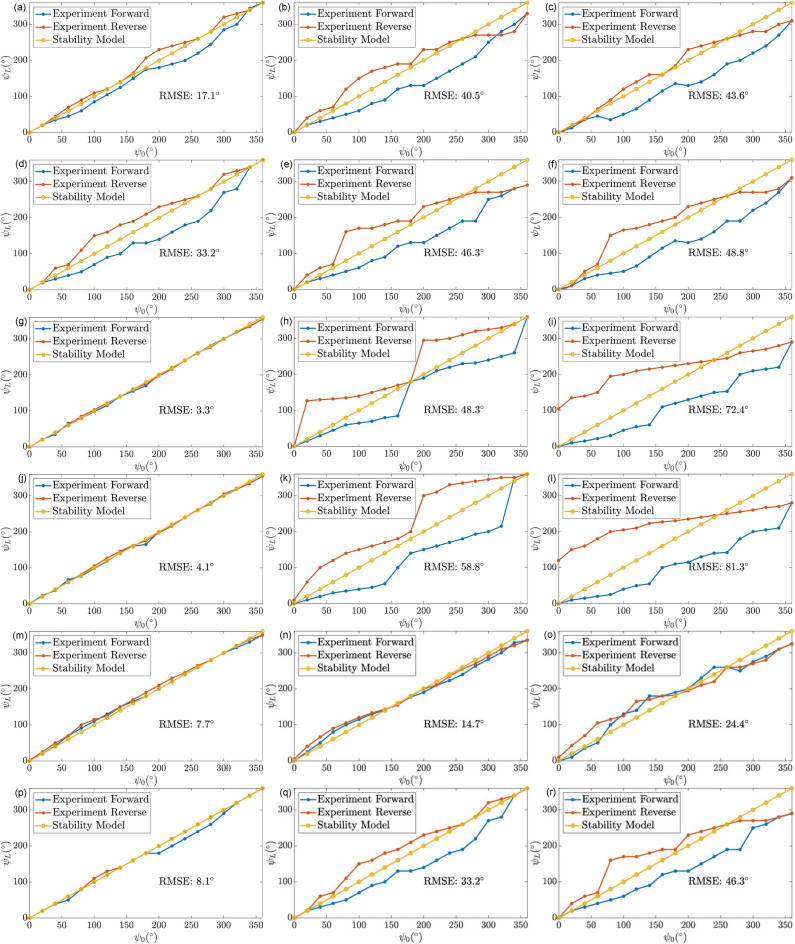
The proximal angle–distal angle correlation of the middle tube with a UAN pattern outer tube for a bending length of 25 mm at tendon strokes of (**a**) 0 mm, (**b**) 1 mm, (**c**) 2 mm, and for a bending length of 50 mm at tendon strokes of (**d**) 0 mm, (**e**) 1 mm, (**f**) 2 mm. The proximal angle–distal angle correlation of the middle tube with a BAN pattern outer tube for a bending length of 25 mm at tendon strokes of (**g**) 0 mm, (**h**) 1 mm, (**i**) 2 mm, and for a bending length of 50 mm at tendon strokes of (**j**) 0 mm, (**k**) 1 mm, (**l**) 2 mm. The proximal angle–distal angle correlation of the middle tube with a QSN pattern outer tube for a bending length of 25 mm at tendon strokes of (**m**) 0 mm, (**n**) 1 mm, (**o**) 2 mm, and for a bending length of 50 mm at tendon strokes of (**p**) 0 mm, (**q**) 1 mm, (**r**) 2 mm.

The results for the $\psi _0$–$\psi _L$ correlation for a UAN outer tube with various tendon strokes of 0 mm, 1 mm, and 2 mm and bending lengths of 25 mm and 50 mm are shown in Fig. 3(a)–(f). The experimental results were compared to the correlations predicted by the model presented in Eq. (11). While the model predicted linear snap-free correlation (see Fig. 3), the experimental results indicated increasing hysteretic behavior in the $\psi _0$–$\psi _L$ correlation with increasing tendon strokes. Since there was non-negligible pre-curvature in the outer tube with UAN pattern, these results indicate there was hysteresis with zero tendon stroke, as shown in Fig. 3(a),(d). Furthermore, minor snapping occurred in these experiments, which was hypothesized to be caused by the presence of friction, and not due to the curvatures of the tubes^26–28^. Increasing the tendon stroke increases the contact force between the two tubes which further contributes towards torsional friction. The RMSE (root-mean-squared error) computed between the experimental results and the model is indicated in the plots.

Similarly, the experiments were conducted with BAN and QSN outer tube geometries, and the results are shown in Fig. 3(g)–(l) and Fig. 3(m)–(r), respectively. The results for the outer tube with BAN pattern exhibited the most hysteresis and consequent snapping behavior as can be seen in Fig. 3(h) and Fig. 3(k). More specifically, the outer tube with BAN pattern had two angles, $0^\circ$ and $180^\circ$, to which the middle tube snapped. In these configurations, the predominant planes of bending containing the notches of the two tubes are adequately aligned. This behavior made the system relatively unstable in the other degrees of rotation, which made this particular outer tube with BAN geometry not ideal for use with the middle tube rotation assembly.

The system with relatively lower snapping behavior was the outer tube with QSN pattern, as shown in Fig. 3(m)–(r). The magnitude of rotational angle changes at the distal end of the robot was not high and mostly ranged between $30^{\circ}\text{ to }40^{\circ }$. The effect of this snapping behavior was further reduced by the outer tube which had significantly higher stiffness than the middle tube. For the QSN pattern experiments, at tendon strokes beyond 1 mm, for $\psi _0 = 360^{\circ }$, $\psi _L < 360^{\circ }$ indicating a decrease in the overall range of $\psi _L$ due to increased friction. More specifically, at a tendon stroke of 1 mm, for $\psi _0 = 360^\circ$, the $\psi _L$ was only $300^\circ$. Therefore, based on the experimental results, tendon stroke values up to 1 mm were deemed to be permissible while rotating the middle tube.

### Kinematic behavior analysis

For the kinematic analysis, outer tubes with UAN and QSN patterns were utilized. We chose to omit the analysis for the BAN geometry, as it was deemed infeasible for the system when investigating the stability of the system with that particular notch geometry. The angle of the plane of bending of the guidewire denoted by $\theta$ (see Fig. 1(d)), is hypothesized to be equal to the middle tube rotation angle, $\psi$. However, due to factors such as gravity, notch patterns of the outer tube, friction, machining errors, and overall bending behavior, $\theta$ might be slightly different from $\psi$. $\theta$ can be estimated using the experimentally obtained values of guidewire curvatures about the *x* and *y* axes using the following equation:

1 $$\begin{aligned} \theta = \text {atan2}(u_y,u_x) \end{aligned}$$

where $u_x$ and $u_y$ are the guidewire curvatures about the *x* and *y* axes taken from the side and top views of the camera setup, respectively.

**Fig. 4:**
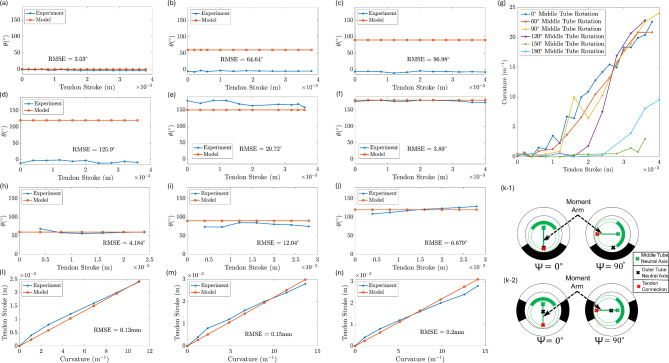
The angle of the plane of bending experiments for a UAN pattern outer tube for middle tube rotation angle of (**a**) $0^{\circ }$, (**b**) $60^{\circ }$, (**b**) $90^{\circ }$, (**d**) $120^{\circ }$, (**e**) $150^{\circ }$, and (**f**) $180^{\circ }$. (**g**) Tendon stroke–curvature correlation of the COAST guidewire robot with a UAN pattern outer tube at middle tube rotations varying from $0\,^\circ,$ to $180\,^\circ.$ The angle of the plane of bending experiments with a QSN pattern outer tube at middle tube rotation angles of (**h**) $60^{\circ }$, (**i**) $90^{\circ }$, and (**j**) $120^{\circ }$. Location of the moment arm with the outer tube with (k-1) UAN pattern and (k-2) QSN pattern when the middle tube is rotated at different angles ($\psi$). The green, black, and red cross marks in the figures are the locations of the middle tube’s neutral axis, outer tube’s neutral axis, and the point of connection of the tendon to the middle tube, respectively. The curvature–tendon stroke model (Eq. (18)) validation for a QSN pattern outer tube with middle tube rotation angle of (**l**) $60^{\circ }$, (**m**) $90^{\circ }$, and (**n**) $120^{\circ }$.

The experimental results are presented in Fig. 4(a)–(n). The experiments were first performed for the guidewire with an outer tube comprising a UAN pattern. The results for the angle of the plane of bending of the guidewire (Fig. 4(a)–(f)) indicate that the assembly does not bend in the intended direction of $\psi$. Note that for these experiments, when the notches of the middle and outer tubes are aligned with a phase difference of 0°, the middle tube rotation angle is measured as 0°.  For example, the RMSE for $\psi = 120^\circ$ and $\psi = 150^\circ$ were approximately $126^\circ$ and $21^\circ$, respectively, as shown in Fig. 4(d),(e). The results indicate that the plane of bending was predominantly in the $0^\circ$ or $180^\circ$ directions. This was because when using an outer tube with a UAN pattern, rotation of the middle tube resulted in the rotation of the moment arm. Assuming the angle between the middle and outer tubes was zero when their notched portions are aligned with a phase difference of $180^{\circ }$ as shown in Fig. 4 (k-1), the location of the neutral axis of the outer tube would not be on the moment arm if the middle tube is rotated to any angle between $0^\circ$ and $180^\circ$ as demonstrated in Fig. 4 (k-1). Under such circumstances, when a moment is applied through a tendon stroke, the direction in which the guidewire will bend will be influenced heavily by the outer tube due to its higher stiffness as compared to the middle tube, resulting in unwanted complexity in the motion of the guidewire.

Furthermore, at $\psi$ values of $150^\circ$ and $180^\circ$, the curvature does not increase as much with higher tendon stroke, as shown in Fig. 4(g), which indicates that the directional preference for bending is near the $0^\circ$ angle of plane of bending of the guidewire, rather than $180^\circ$. This is because the directional stiffness for the guidewire in the bending direction is increased at $150^\circ$ and $180^\circ$, so more tendon stroke is needed to bend the guidewire when positioned in those directions. For the middle tube rotation angles less than $120^\circ$, the tendon stroke—curvature relationship was fairly linear, with the tendon stroke required to reach higher curvatures increasing with increasing middle tube rotation angles, as the notches would get misaligned the further the middle tube rotates from $0^\circ$.

The angles of plane of bending of the guidewire for the outer tube with a QSN pattern are shown in Fig. 4(h)–(j). This is hypothesized to stay constant throughout the bending with higher tendon strokes, however, due to friction interactions and gravity, the values tend to deviate from the model, with a maximum RMSE of $12.04^\circ$ for the middle tube rotation angle of $90^\circ$, which translated to approximately 3.34% with respect to the overall actuation range of middle tube rotation (0–$360^{\circ }$). The behavior was consistent with the modeling hypothesis for rotation: The neutral axis of the outer tube with QSN joint coincides with its central axis. As a result, the outer tube’s neutral axis would lie on the moment arm irrespective of the orientation of the middle tube with respect to the outer tube (see Fig. 4 (k-2)). Consequently, the bending direction of the guidewire would be equal to the relative angle between the middle and outer tubes, resulting in a relatively simpler mechanism. Based on the stability and kinematic analysis for the outer tube with three different notch geometries, we choose the QSN geometry as the optimal geometry for the middle tube rotation. The choice of the notch parameters was guided by previous work, which found optimal parameters for the COAST guidewire^17,18^. If the depth of cut is too shallow, the guidewire would demonstrate an undesirable increase in stiffness. Conversely, if the depth of cut is too high, the guidewire would be relatively more susceptible to failure.

Using the camera setup and image processing, the curvature of the guidewire which utilized an outer tube with QSN geometry, was measured at middle tube rotation angles of $60^{\circ }$, $90^{\circ }$, and $120^{\circ }$, and for various tendon strokes up to 2.8 mm. The bending length was maintained at 50 mm for the experiments. Results depicting the estimated tendon stroke of the guidewire using the model in Eq. (18) and the applied tendon strokes are presented in Fig. 4(l), Fig. 4(m), and Fig. 4(n) for $60^{\circ }$, $90^{\circ }$, and $120^{\circ }$ rotation angles, respectively. The maximum RMSE for the model compared to the experimental results was 0.2 mm, for a rotation angle of $120^\circ$, which translated to approximately 7.14% with respect to the overall actuation range. The model exhibited an average RMSE of 0.16 mm based on 0.13 mm, 0.15 mm, and 0.2 mm RMSE of tendon strokes for middle tube rotation angles of  60°, 90°, and 120°, respectively.﻿﻿ This indicated that the model was adequately accurate in predicting the required tendon strokes for the guidewire.

### Navigation experiment results

**Fig. 5:**
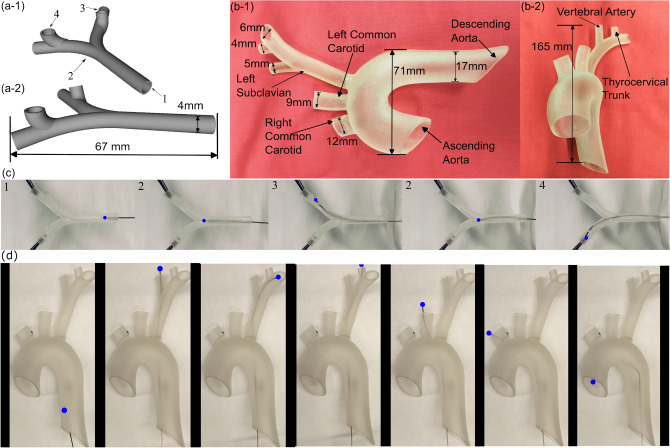
(**a-1**) and (**a-2**) Different viewpoints of the rendering of the custom phantom vessel with labeled locations. (**b-1**) and (**b-2**) Different viewpoints of the aortic arch phantom vessel with labeled locations. (**c**) Images of the demonstration of the guidewire passing through several locations along the custom phantom; the numbers refer to the location of the guidewire as labeled in (**a-1**). (**d**) Images of the demonstration of the guidewire passing through several locations along the flexible aortic arch phantom. The blue dots refer to the position of the tip of the guidewire.

A custom model of a mock vascular structure was created to show an example of a geometry that the guidewire can navigate. Fig. 5(a-1) and Fig. 5(a-2) show renderings of this phantom. This model was 3D-printed using a Form 3 printer (Formlabs, Somerville, MA, USA) with clear resin, and consists of some confined volumes that can be traversed using teleoperation, using a programmable remote controller. The inner diameter of the mock vasculature was 4.24 mm, with the initial straight segment of a length extending for approximately 33 mm, and the right and left exit points being approximately 17 mm from the initial straight segment. The exit points 3 and 4 were facing up about 7 mm above the initial horizontal path, positioned perpendicular to the entry point 1.

In addition, a phantom model of the aortic arch was made using Stratasys flexible resin and was 3-D printed on a Stratasys J35 printer using a combination of the VeroUltraClear and ElasticoClear materials, as shown in the images in Fig. 5(b-1) and Fig. 5(b-2). The phantom is based on de-identified CT scan data of a human aortic arch. It shows the ascending and descending aortas, the left subclavian artery, and the left and right common carotid arteries. The inner diameter of the descending aorta branch is approximately 17 mm, whereas the inner diameter of the common carotid and subclavian branches is much smaller, with the dimensions shown in Fig. 5(b-1) and Fig. 5(b-2). The phantom was relatively flexible as shown in the supplementary video.

The control strategy described in Algorithm 1 was implemented to smoothly navigate the guidewire with a QSN geometry for the outer tube through the custom phantom while reducing the effects of instabilities. For the navigation demonstration, the guidewire was directed towards a bifurcation point, which was point 2 as labeled in Fig. 5(a-1) and Fig. 5(a-2), and then directed towards each of the exit points, 3 and 4, each requiring the middle tube to be rotated. The notches of the middle tube of the guidewire were initially pointed towards the right, and the guidewire was first navigated towards point 2 with a forward motion. Then, the tendon was pulled to navigate the guidewire towards the right branch followed by a middle tube rotation of approximately $90^\circ$ counterclockwise to navigate the guidewire to point 3. Then, the guidewire was retracted back to point 2, and the tendon was relaxed. The middle tube was then rotated $180^\circ$ clockwise, and the tendon was pulled while the guidewire was fed into the left branch. This was followed by the middle tube rotation of $90^\circ$ clockwise to exit out of point 4. Images of the guidewire during the navigation experiments taken at the key locations are shown in Fig. 5(c).

Similarly, the control strategy was also used to navigate the anatomically similar phantom model of the aortic arch, with the images of the navigation experiment shown in Fig. 5(d). The guidewire is first rotated and advanced towards the left subclavian branch and navigated towards the vertebral and thyrocervical trunk branches. Subsequently, the guidewire is retracted, then rotated and bent to advance towards the left common carotid artery branch, and the steps are repeated for the right common carotid and the ascending aorta branches. While these navigation experiments were generally successful and encountered no major challenges, it may not be possible to traverse all different anatomies with these specific guidewire parameters. For example, in another version of the aortic arch phantom with more branching, we found it difficult to navigate to the furthermost branch point of the left and right common carotid artery. Hence, we cut further bifurcation branches of the right common carotid artery and reduced the length of the left common carotid artery to arrive at the aortic arch phantom shown in Fig. 5(b-1) and Fig. 5(b-2)—we used this phantom to demonstrate the steerability of the guidewire. Thus, it is important to note that for relatively more complicated anatomy with more branching and tortuosity, it may be necessary to choose different guidewire parameters to ensure its navigability to the target location. Further studies are planned to test this guidewire system under fluid flow to determine its stability in potential endovascular procedures.

## Methods

### Stability analysis

#### Derivation

The rotation of the middle tube with respect to the outer tube was expected to introduce snapping behavior due to elastic instabilities which is a common problem in concentric tube robots^25,29^. Concentric tube robots snap due to elastic instabilities, where tubes rapidly release stored strain energy, causing them to jump from one configuration to another^30^. This can be undesirable as it can decrease the workspace of the robot at certain rotation angles. To study these snapping effects from the middle tube rotation of the COAST guidewire, we model the system as a concentric tube robot with static equilibrium conditions for a Cosserat rod applied to each tube^25,29,31^. The expected outcome of the stability analysis was to design a configuration that had the least predicted elastic instabilities. In this segment, we briefly overview the equations governing the standard mechanics model of a concentric tube robot. The equations are as follows:

2 $$\begin{aligned} \begin{aligned}&\dot{n_i} + f_i = 0 \\&\dot{m_i} + \dot{p_i} \times n_i + l_i = 0 \end{aligned} \end{aligned}$$

with the kinematic equations for the motion shown as

3 $$\begin{aligned} \begin{aligned}&\dot{p_i} = R_i \hat{z}\\&\dot{R_i} = R_i \hat{u_i} \end{aligned} \end{aligned}$$

where *i *is the number of the tube in the robot assembly, *n* is the internal forces,  *f*  is the external distributed forces, *m* is the internal moment of the tube, *p* is the position of the tubes, *l* is the external distributed moment, *R* is the orientation of the tubes, and *u *is the curvature vector for the tube, where all the variables are functions of the common centerline of the tubes along the arclength *s*$\in$ [0 *L*]. As shown in^29^, the dot operator,[$\dot{{\phantom{a}}}$], denotes the derivative with respect to the arclength, and the hat operator^32^, [$\hat{{\phantom{a}}}$], represents the vector’s skew-symmetric form.

The stability regions of the COAST guidewire robot were modeled using the Kirchoff kinetic energy analogy, which can be described as the stored elastic energy of a slender rod that undergoes bending and torsion, but not elongation or shear^26^. It is noted that the following analysis assumes zero torsional friction between the tubes. Furthermore, it is assumed that the distance between the centerlines of the tubes is negligible. The stiffness matrix, $K_j$, for each tube is given by:

4 $$\begin{aligned} K_j&= \begin{bmatrix} E_jI_{xx,j} & 0 & 0 \\ 0 & E_jI_{yy,j} & 0 \\ 0 & 0 & G_jJ_{j} \end{bmatrix} \end{aligned}$$

where $E_j$ is the Young’s modulus of the *j*^th^ tube, $G_j$ is the rigidity modulus of the *j*^th^ tube, and $I_{xx,j}$, $I_{yy,j}$, and $J_{j}$ are the second moment of area about the *x*, *y*, and *z*-axes of the *j*^th^ tube respectively. The rotation matrix, $R_j$, for each tube is given by:

5 $$\begin{aligned} R_j&= \begin{bmatrix} \cos {\psi _j} & -\sin {\psi _j} & 0 \\ \sin {\psi _j} & \cos {\psi _j} & 0 \\ 0 & 0 & 1 \end{bmatrix} \end{aligned}$$

where $\psi _j$ is the rotation about the *z*-axis of the *j*^th^ tube with respect to the fixed frame of reference. It is assumed that the curvature of the two tubes only occurs in the *x*-direction. Therefore, the initial curvature of each individual tube, $u_j^*(s)$, can be expressed as:

6 $$\begin{aligned} u_j^*(s) = \begin{bmatrix} {u^j}_x&0&0 \end{bmatrix}^{T} \end{aligned}$$

where $u^j_x$ is the magnitude of the curvature in the x-direction for the *j*^th^ tube. The combined stiffness of the tubes is denoted as *K*, where $K = \sum _{j=1}^{2} R_j K_j R_j^T$. To combine the two curvatures and obtain an overall curvature of the robot $u_B$, the following relationship is utilized:

7 $$\begin{aligned} u_B = K^{-1}(R_1 K_1 u^*_1 + R_2 K_2 u^*_2) \end{aligned}$$

This overall curvature is used to determine the modified curvature when the middle tube rotates. The total energy for the two tubes can be represented as the sum of the energy of the tubes with respect to its change in curvature namely:

8 $$\begin{aligned} T[u_1,u_2] = \sum _{j=1}^{2} \int _{\beta }^{L} \Delta u_j^{T} K_j \Delta u_j ds_j \end{aligned}$$

where *s* is the arclength parameter, *L* is the overlapped length of the tubes, *T* is the energy of the system, and $\Delta u_j = u_j - u_j^*$. The arclength when $s=0$ is defined as the point where the rotation begins for both tubes. Using the convention of previous approaches^26^, $\beta$ is defined as the distance between the actuation point of the outer and middle tubes where $\beta \le 0$. $u_j$ can be algebraically related to $u_B$ through $\psi _j$ and the energy functional *T* can be written as a function of $\psi _j$ for $j = 1,2$. The energy functional can then be used in conjunction with the Euler–Lagrange equations to obtain the kinematics of the system^26^.

Consequently, the spatial configuration of the robot is given by the boundary value problem (BVP) with states in $\psi _j$, $G_j J_j \psi _j'$, $p_B$ (position vector of the robot), and $R_B$. The corresponding differential equations that govern the kinematics can be described as:

9 $$\begin{aligned} \begin{aligned}&(G_j J_j \psi _j ')' = -u^T_B K_j \frac{\partial { R_{\psi _j}}}{\partial { \psi _j}} u^*_j\\&p'_B = R_B \hat{z}\\&R'_B = R_B \hat{u_B} \end{aligned} \end{aligned}$$

with the boundary conditions given as

10 $$\begin{aligned} \begin{aligned}&p_B(0) = 0\quad R_B(0) = I \\&\psi _j(\beta ) = \psi _{\beta , j} \quad (G_j J_j \psi _j ')(L) = 0 \end{aligned} \end{aligned}$$

Using these equations and the equation of the relative tube angle of $\alpha = \psi _1 - \psi _2$, where $\psi _1$ and $\psi _2$ are related to the middle tube and outer tube, respectively, the variation of $\alpha$ over the arc length, *s*, can be derived to be:

11 $$\begin{aligned} \alpha {''} = U_x^T A(\alpha ) U_x \sin \alpha \end{aligned}$$

where $U_x\,= [u_x^1\,\,u_x^2]^T$. Consider the stiffness in the x, y, and z directions are defined as follows:

12 $$\begin{aligned} \begin{aligned} \zeta _j&= E_jI_{xx,j}\\ \sigma _j&= E_jI_{yy,j}\\ \eta _j&= G_jJ_{j} \end{aligned} \end{aligned}$$

The matrix A($\alpha$) in Eq. (11) is given as

13 $$\begin{aligned} & \tau = (\sigma _1 + \sigma _2) \begin{bmatrix} \zeta _1 \zeta _2 + \zeta _1 \sigma _1\\ (\zeta _2 \sigma _1 - \zeta _1 \zeta _2)\cos (\alpha ) \end{bmatrix} \begin{bmatrix} (\zeta _1 \sigma _2 - \zeta _1 \zeta _2)\cos (\alpha )\\ \zeta _1 \zeta _2 + \zeta _2 \sigma _2 \end{bmatrix}^T \nonumber \\ & \upsilon = \frac{\eta _1 \eta _2}{\eta _1+\eta _2} \begin{vmatrix} \zeta _1 + \sigma _2&(\zeta _1 - \sigma _1)\cos (\alpha )\\ (\zeta _2 - \sigma _2)\cos (\alpha )&\sigma _1 + \zeta _2 \end{vmatrix}^2 \nonumber \\ & A(\alpha ) = \frac{\tau }{\upsilon } \end{aligned}$$

The boundary conditions as shown in^25,26^ are defined as $\alpha (0) = \alpha _0$ and $\alpha '(L) = 0$.

It has been shown that tubes can be laser machined to reduce the ratio of bending to torsional stiffness, which improves the stability^33,34^. Similarly, for this study, the outer tubes with notch patterns, which may ensure elastic stability in all rotation angles of the middle tube, were utilized. Snapping instabilities can still occur if high curvatures are employed. Therefore, methods for the design and snap prediction will still be needed. The stability of the COAST guidewire robot is simulated to ensure the robust operation of the robot at any reasonable curvatures and lengths needed for surgical procedures. We solve the BVP presented in Eq. (11) to obtain a correlation between $\alpha (0)$ and $\alpha (L)$ with a shooting method in MATLAB R2023a using the ode45 function, a widely used numerical integration method which is built upon the principles of an explicit Runge-Kutta (4,5) formula, specifically the Dormand-Prince pair^35^. This solver stands out as a single-step algorithm^36^, indicating that when it calculates the value of *y* at a given time point $t_n$, it exclusively depends on the solution obtained at the immediately preceding point $t_{n-1}$.

To calculate the stiffness of the tubes with different notch geometries for the simulations, the second moment of area for the different notch geometries must be determined. For the UAN pattern, the procedure presented in^16^ was utilized, and for the BAN joint, the second moment of area about the three axes was numerically computed using the analysis presented in^37^.

**Fig. 6:**
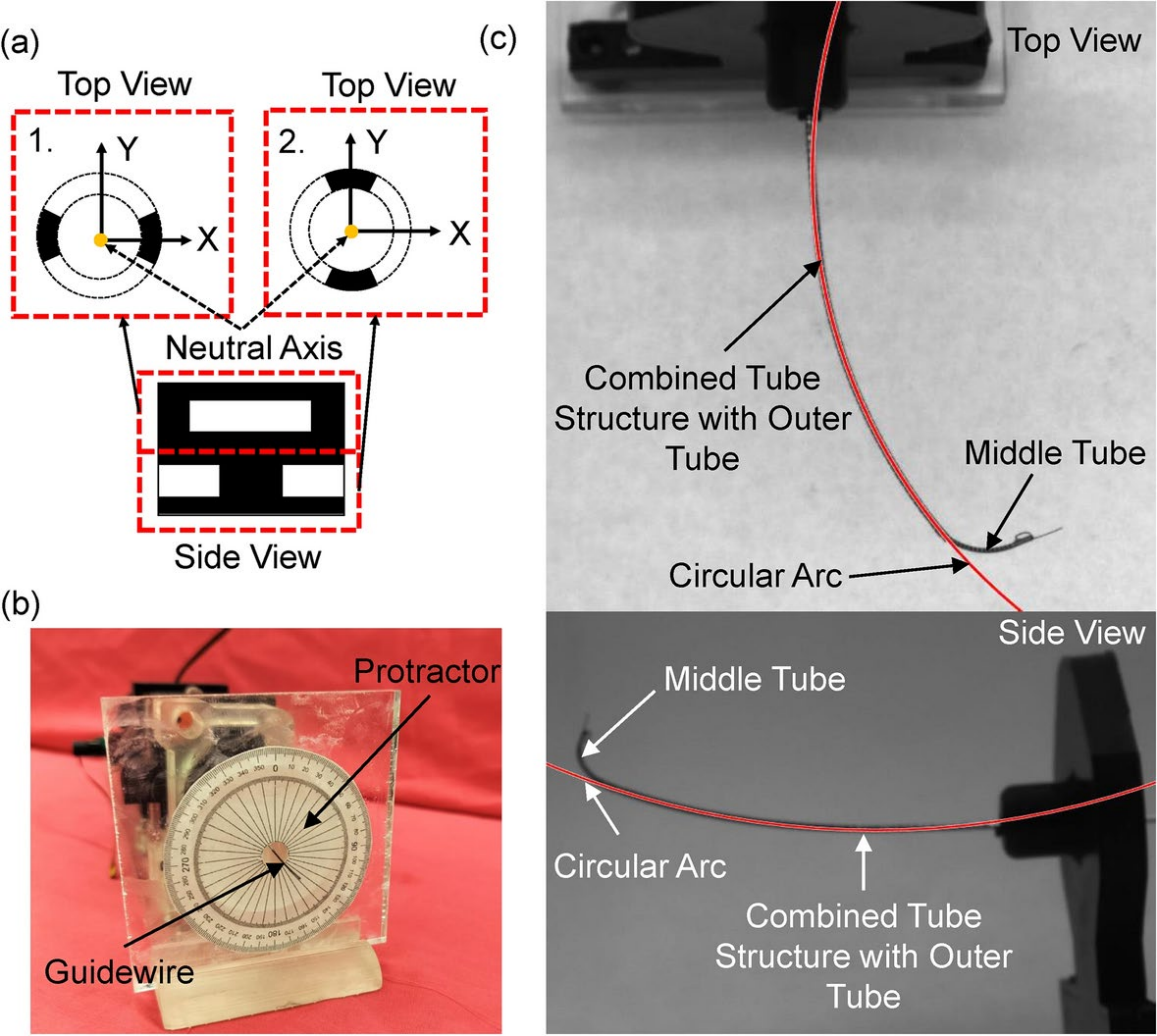
(**a**) Top and side views of one quad-directional symmetric notch (QSN) unit with labeled neutral axes. (**b**) Image of protractor attached to an acrylic plate used to capture the proximal angle–distal angle correlation of the middle tube. (**c**) Top and side views of the combined tube structure used for calculating the curvatures for the kinematic experiments with circular arcs overlaid on the image to show the curvature calculation for the combined tube structure.

The procedure to determine the second moment of area of the QSN joint is detailed below. Consider a unit of the outer tube with the QSN pattern as shown in Fig. 6(a). The unit consists of two BSN joints attached in series with a phase difference of $90^{\circ }$. Due to this phase shift between the two joints, it was assumed that when a moment is applied about a principal axis, depending on the axis, only one of the joints will bend. Based on the orientation of the frame of reference attached to the outer tube (Fig. 6(a)), the second moment of area of the two joints about their neutral axis (which is aligned with their central axis) are derived as follows:

14 $$\begin{aligned} & \begin{aligned} I_{xx, up}&= I_{yy, low}&=(r_{o}^4 - r_{i}^4)\frac{\phi - \sin \phi }{4} \ \end{aligned} \end{aligned}$$

15 $$\begin{aligned} & \begin{aligned} I_{xx, low}&=I_{yy, up}&= (r_{o}^4 - r_{i}^4)\frac{\phi + \sin \phi }{4} \ \end{aligned} \end{aligned}$$

where $r_{o}$ and $r_{i}$ are the outer and the inner radius of the outer tube respectively, $\phi$ is the central angle of the tube created by the laser cut. The subscripts {*up*, *low*} of the second moment of area denote the upper and lower joints respectively. For a given depth of cut, it can be shown that $I_{yy, up}>> I_{xx, up}$ and $I_{xx, low}>> I_{yy, low}$. Therefore, it is assumed that when a moment is applied about the *x*-axis of the outer tube, only the upper joint will bend, and when a moment is applied about the *y*-axis of the tube, only the lower joint will bend. The second moment of area matrix of the QSN tube can be written as:

16 $$\begin{aligned} I_{QSN} = \begin{bmatrix} I_{xx, up} & 0 & 0\\ 0 & I_{yy,low} & 0\\ 0 & 0 & J_{QSN} \\ \end{bmatrix} \end{aligned}$$

where $J_{QSN} = I_{xx, up} + I_{yy, low}$. From Eq. (14) it is noted that $I_{xx,up} = I_{yy,low}$. Since the system is designed such that only the middle tube can be rotated, $\alpha = \psi _1$, in Eq. (11). For simplicity, $\psi _1$ will be denoted as $\psi$ for the remainder of the paper. Therefore $\alpha = \psi$.

#### Experimental procedure

To measure $\psi _L$ of the middle tube, a protractor was utilized. The protractor was attached to a plate near the distal end of the outer tube, with the middle tube slightly protruding from the combined tube structure at the distal end to accurately ascertain the rotational motion, as shown in Fig. 6(b). The middle tube was initially positioned such that the rotational angles at its proximal and distal ends are at $0^\circ$. The middle tube was then rotated at the proximal end with varying $\psi _0$ in increments of $20^\circ$. The proximal end of the middle tube was rotated until $360^\circ,$ and then rotated back to $0^\circ$ also in decrements of $20^\circ$. Three outer tubes with UAN, BAN, and QSN patterns respectively, were utilized for the experimental study. The notch geometries of these tubes are presented in Table 2. The notch geometries were selected based on the simulation study to lie in the stable regions of the design space.

### Kinematic analysis

#### Model derivation

To derive the relationship between the tendon stroke, $X_1$, and curvature, $\kappa$, of the guidewire with a QSN joint as the outer tube, the effect of middle tube rotation needs to be captured. The second moment of area matrix of the outer tube can be determined by using Eqs. (14)–(15). When the middle tube is rotated by an angle of $\psi$, assuming the rotation has propagated uniformly about the length of the middle tube, in the frame of reference attached to the middle tube, the outer tube would be rotated in the opposite direction with the same magnitude ($-\psi$). To compute the elongation of the tendon, the second moment of area matrix of the outer tube, $I_{out}$ needs to be transformed. However, since the second moment of area of the outer tube about the *x* and *y* axes are equal, the transformation would not change $I_{out}$. Mathematically this can be written as:

17 $$\begin{aligned} R_{z,-\psi }I_{out}R_{z,-\psi }^T = I_{out} \end{aligned}$$

where, $R_{z,-\psi }$ is the rotation matrix about *z*-axis with a magnitude of $-\psi$.

For the kinematic model, only the *x*-component of the transformed $I_{out}$ matrix is required, which is the first element of the $I_{out}$ matrix. The model, first presented in^16^, defines the tendon stroke $X_1$ as a function of the guidewire curvature $\kappa$ as a summation of two terms: (1) the kinematic term and (2) the elongation term. The model is presented below:

18 $$\begin{aligned} X_1 = \underbrace{\Delta L^{kin}(\kappa , X_b)}_\text {{Kinematic Term}} + \underbrace{\frac{E(I_{xx, out} + I_{xx, mid})L_{total}}{\Delta y E_t\pi r_t^2}\kappa }_\text {{Tendon Elongation Term}} \end{aligned}$$

In Eq. (18), $X_b$ is the bending length of the guidewire, $L_{total}$ is the length of the tendon, $\Delta y$ is the length of the moment arm (distance between the neutral axis of the middle tube and the attachment point of the middle tube and the tendon), $E_t$ is the Young’s modulus of the tendon, $I_{xx,mid}$ is the second moment of area of the middle tube, and $r_t$ is the radius of the tendon. It is noted that $I_{xx, out}$ is derived using Eq. (14).

#### Experimental procedure

To examine the kinematic relationship for the curvature–tendon stroke at different middle tube rotation angles, the curvature of the guidewire was measured from the top and side at multiple tendon stroke values and multiple rotations of the middle tube. To measure the curvature of the guidewire, two high-resolution CMOS cameras (Zelux™ 1.6 *MP*, Thorlabs Inc., NJ, United States) were strategically positioned perpendicular to each other, with their lenses focused on the bending segment of the guidewire. Before actuating the tendon, the middle tube was first rotated to the appropriate rotation angle, $\psi$. Then, the tendon stroke was increased in increments of 0.4 mm to achieve higher curvatures for the guidewires. Examples of images of the guidewire with 2.8 mm tendon stroke and $120^\circ$ middle tube rotation is shown in Fig. 6(c) with the top view and side view.

The experimental setup had a small section of the middle tube outside of the combined tube structure at the distal end to visually ensure rotation was taking place, and a series of image processing steps were taken to measure the curvature in the images. First, the image was cropped and edge detection^38^ was performed on the image. Then, the connected components smaller than 40 pixels were filtered out. Subsequently, the image was inverted and morphologically eroded to close some segments. This process effectively binarized and captured only the combined tube structure in the image while filtering out other objects, including the middle tube protruding out. After this step, a best-fit circle^39^ was fit to the binarized image of the combined tube structure to find the curvature of the tube. Sample results of the best-fit circle overlaid on top of the original image are shown in Fig. 6(c).

### Control strategy

It is noted that the angle of the bending plane, $\theta$, is measured with respect to the fixed frame of reference attached to the outer tube. Let $\kappa _d$ and $\theta _d$ be the desired curvature and desired angle of the bending plane , respectively, and $\kappa _0$ and $\theta _0$ be the initial curvature and angle of the bending plane, respectively. The initial curvature is due to the actuation of the tendon and not the pre-curvature.

The snap-free region of the guidewire will be defined as a function of its curvature, $\kappa$. In addition, since the curvature of the guidewire is a function of the tendon actuation, the snap-free region of the guidewire will also be defined as a function of tendon actuation $X_t$. Let $\Phi$ be the snap-free region in the guidewire curvature/tendon actuation range determined experimentally. The control algorithm can be defined as follows:

**Algorithm 1:**
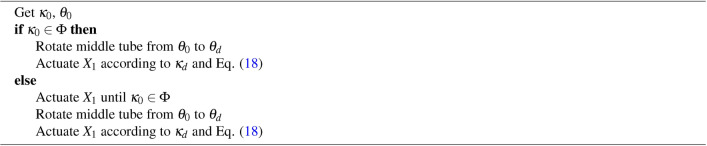
Control algorithm

As demonstrated by the results, the contribution of friction towards the snapping behavior may exacerbate at higher curvatures of the middle tube which can be controlled through the tendon stroke. A model to predict snapping by considering the friction between the tubes can be derived, however, that is beyond the scope of this study. The experimental results were used to physically determine the snap-free regions in the actuation range of the guidewire. Based on the experimental results, tendon stroke values up to 1 mm were deemed to be permissible while rotating the middle tube. This information was used to control the guidewire such that the guidewire bent in a desirable plane while avoiding snapping.

## Conclusion and future work

This manuscript outlines the creation and assessment of a robotically steerable guidewire system that integrates the COaxially Aligned STeerable (COAST) actuation mechanism with a novel middle tube rotation feature. Simulation study for different notch geometries of the outer tube was conducted to determine the optimal design for stable rotation of the middle tube. Experiments were performed on different notch geometries to determine the optimal notch geometry of the outer tube for bending the system at various middle tube rotation angles. While the stability model predicted a linear snap-free behavior, experiments showed that the guidewire system exhibited minor snapping behavior. This is hypothesized to occur due to friction between the tubes. Using these experiments, a control strategy was devised to minimize snapping caused due to friction in the system. The motion capabilities of the robotic system were evaluated using a custom phantom model and an aortic arch phantom model. The guidewire system with the optimal notch geometry was successfully navigated through the bifurcation paths of both phantom models, highlighting the efficacy of this mechanism. For relatively more complicated anatomy with more branching and tortuosity, it may be necessary to choose different guidewire parameters to ensure its navigability to the target location. Future endeavors will focus on assessing the functionality of the system within anatomical models with fluid flow and evaluating the capability of this guidewire system to navigate through animal vasculature. The development of a robust control strategy for achieving high curvatures while incorporating friction interactions between the tubes will also be considered as part of our future work.

## Supplementary Information

Supplementary Information.

## Data Availability

The datasets used and/or analyzed during the current study are available from the corresponding author on reasonable request.
